# Supplementary material for: Picotesla-sensitivity microcavity optomechanical magnetometry
Source: Light Sci Appl. 2024 Sep 29;13:279. doi: 10.1038/s41377-024-01643-7 (PMC11439073; doi:10.1038/s41377-024-01643-7)
Supplement: Supplementary file 1 — Supplementary Information for “Picotesla-sensitivity microcavity optomechanical magnetometry” [file 41377_2024_1643_MOESM1_ESM.pdf]

Supplementary Information for "Picotesla-sensitivity microcavity optomechanical magnetometry"

Zhi-Gang Hu[1,2,†], Yi-Meng Gao[1,2,†], Jian-Fei Liu[1,2,†], Hao Yang[1,2], Min Wang[1,2], Yuechen Lei[1,2], Xin Zhou[1,2], Jincheng Li[1,3], Xuening Cao[1,2], Jinjing Liang[1,2], Chao-Qun Hu[1,2], Zhilin Li[1,4], Yong-Chang Lau[1,2,††], Jian-Wang Cai[1,2,∗∗], and Bei-Bei Li[1,2,4,∗]

[1] Beijing National Laboratory for Condensed Matter Physics, Institute of Physics, Chinese Academy of Sciences, Beijing 100190, China

[2] University of Chinese Academy of Sciences, Beijing 100049, China

[3] School of Physics, Beihang University, Beijing 100191, China

[4] Songshan Lake Materials Laboratory, Dongguan 523808, Guangdong, China

† These authors contributed equally to this work.

†† yongchang.lau@iphy.ac.cn; ∗∗ jwcai@iphy.ac.cn; ∗ libeibei@iphy.ac.cn

S1. Noise and sensitivity analysis

To investigate the relationship between the sensitivity and the optical (mechanical) parameters of the MCOM, we conduct a comprehensive analysis of the noise sources in the optomechanical sensing system and derive the expression of sensitivity. Here we consider the intensity modulation readout mechanism while taking into account the laser frequency detuning. The linearized Hamiltonian of the system can be expressed as follows:

$$H = -\frac{\hbar\Delta}{2}(X^2+Y^2) + \frac{\hbar\Omega_\mathrm{m}}{2}(q^2+p^2) + 2\hbar g X q \tag{1}$$

Here, $\hbar$ is the reduced Planck constant. Δ represents the frequency detuning between the laser frequency ω_L and the cavity resonacne ω_o, giving by $\Delta = \omega_\mathrm{L} - \omega_\mathrm{o}$. The variables X and Y correspond to the optical amplitude and phase quadratures, respectively. Here, $\sqrt{X^2+Y^2}$ is the amplitude of the light, and $\arctan(\frac{Y}{X})$ is the phase of the light. Ω_m denotes the angular frequency of the mechanical mode. q and p represent the dimensionless mechanical position and momentum normalized by $x_\mathrm{zp} = \sqrt{\frac{\hbar}{2m_\mathrm{eff}\Omega_m}}$ and $p_\mathrm{zp} = \sqrt{\frac{\hbar m_\mathrm{eff}\Omega_\mathrm{m}}{2}}$. Here x_zp and p_zp are zero-point displacement and momentum, respectively, with m_eff denoting the effective mass of the mechanical mode, respectively. The cavity-enhanced optomechanical coupling rate is given by $g = g_0\sqrt{N}$, where g_0 is the single-photon optomechanical coupling rate defined as $g_0 = G_\mathrm{om} x_\mathrm{zp}$. Here, $G_\mathrm{om} = \frac{d\omega_\mathrm{o}}{dR}$ is the optomechanical coupling coefficient, which quantifies how much ω_o shifts with the radius (R) change. N represents the steady-state photon number in the cavity, given by $N = \frac{\kappa_1 N_\mathrm{in}}{\Delta^2+\frac{\kappa^2}{4}}$, where N_in is the input photon number per second. The equations of motion of the system can be obtained using the quantum Langevin equation:

$$\dot{X} = -\Delta Y - \frac{\kappa}{2}X + \sqrt{\kappa}X_\mathrm{in} \tag{2a}$$

$$\dot{Y} = \Delta X - \frac{\kappa}{2}Y - 2gq + \sqrt{\kappa}Y_\mathrm{in} \tag{2b}$$

$$\dot{q} = \Omega_\mathrm{m} p \tag{2c}$$

$$\dot{p} = -\Omega_\mathrm{m} q - \gamma p + \sqrt{2\gamma}p_\mathrm{in} - 2gX \tag{2d}$$

In these equations, κ represents the total energy decay rate of the optical mode, given by $\kappa = \kappa_0 + \kappa_1$, with κ_0 and κ_1 denoting the intrinsic and external energy decay rates, respectively. X_in and Y_in are the operators representing the optical quadrature fluctuations, given by $X_\mathrm{in} = \sqrt{\frac{\kappa_0}{\kappa}}X_0 + \sqrt{\frac{\kappa_1}{\kappa}}X_1$ and $Y_\mathrm{in} = \sqrt{\frac{\kappa_0}{\kappa}}Y_0 + \sqrt{\frac{\kappa_1}{\kappa}}Y_1$. Here, X_0 and Y_0 describe the vacuum fluctuations from the thermal bath, while X_1 and Y_1 describe the fluctuations from the laser. For coherent light, the power spectral densities (PSDs) of the optical quadrature fluctuations are given by:

$$S_{X_\mathrm{in}X_\mathrm{in}} = S_{X_0X_0} = S_{X_1X_1} = \frac{1}{2} \tag{3a}$$

$$S_{Y_\mathrm{in}Y_\mathrm{in}} = S_{Y_0Y_0} = S_{Y_1Y_1} = \frac{1}{2} \tag{3b}$$

$$S_{X_\mathrm{in}Y_\mathrm{in}} = S_{X_0Y_0} = S_{X_1Y_1} = 0 \tag{3c}$$

γ is the energy decay rate of the mechanical mode. p_in is the mechanical momentum fluctuation operator. For symmetrized bath PSD and at high-temperature limit,

$$S_{p_\mathrm{in}p_\mathrm{in}} = \frac{k_B\mathcal{T}}{\hbar\Omega_\mathrm{m}} \tag{4}$$

Here, k_B is the Boltzmann constant and $\mathcal{T}$ is the temperature.

The Eqs. (2) can be solved straightforwardly in the frequency domain. Taking the Fourier transform, we obtain the steady-state solutions

$$X(\Omega) = \frac{2g\Delta}{(\kappa/2-i\Omega)^2+\Delta^2}q + \frac{\sqrt{\kappa}(\kappa/2-i\Omega)}{(\kappa/2-i\Omega)^2+\Delta^2}X_\mathrm{in} - \frac{\sqrt{\kappa}\Delta}{(\kappa/2-i\Omega)^2+\Delta^2}Y_\mathrm{in} \tag{5a}$$

$$Y(\Omega) = \frac{-2g(\kappa/2-i\Omega)}{(\kappa/2-i\Omega)^2+\Delta^2}q + \frac{\sqrt{\kappa}\Delta}{(\kappa/2-i\Omega)^2+\Delta^2}X_\mathrm{in} + \frac{\sqrt{\kappa}(\kappa/2-i\Omega)}{(\kappa/2-i\Omega)^2+\Delta^2}Y_\mathrm{in} \tag{5b}$$

$$q(\Omega) = \chi_\Delta[\sqrt{2\gamma}p_\mathrm{in} - 2\sqrt{C_\mathrm{eff}^\Delta\gamma}\frac{(\kappa/2-i\Omega)^2}{(\kappa/2-i\Omega)^2+\Delta^2}X_\mathrm{in} + 2\sqrt{C_\mathrm{eff}^\Delta\gamma}\frac{(\kappa/2-i\Omega)\Delta}{(\kappa/2-i\Omega)^2+\Delta^2}Y_\mathrm{in}] \tag{5c}$$

Here, Ω is the Fourier frequency, $\chi_\Delta(\Omega)=\frac{\Omega_\mathrm{m}}{-\Omega^2-i\Omega\gamma+\Omega_\mathrm{m}^2+\frac{4g^2\Omega_\mathrm{m}\Delta}{(\kappa/2-i\Omega)^2+\Delta^2}}$ is the modified mechanical susceptibility in the presence of the optical field, $C_\mathrm{eff}^\Delta(\Omega)=\frac{4g^2}{\kappa\gamma(1-2i\Omega/\kappa)^2}$ is the effective optomechanical cooperativity. The output optical quadratures can be obtained using the input-output relations $X_\mathrm{out}=X_1-\sqrt{\kappa_1}X$ and $Y_\mathrm{out}=Y_1-\sqrt{\kappa_1}Y$ under any coupling condition. Here, we define $\kappa_0=\eta\kappa_1$, where $\eta>1,\eta=1,\eta<1$ denote the under-coupled, critical-coupled, and over-coupled conditions, respectively. The output optical quadratures are given by:

$$\begin{aligned}X_\mathrm{out}(\Omega)=&-\frac{2g\sqrt{\kappa_1}\Delta}{(\kappa/2-i\Omega)^2+\Delta^2}q+\frac{(\frac{\eta-1}{2}\kappa_1-i\Omega)(\kappa/2-i\Omega)+\Delta^2}{(\kappa/2-i\Omega)^2+\Delta^2}X_1-\frac{\sqrt{\eta}\kappa_1(\kappa/2-i\Omega)}{(\kappa/2-i\Omega)^2+\Delta^2}X_2\\&+\frac{\kappa_1\Delta}{(\kappa/2-i\Omega)^2+\Delta^2}Y_1+\frac{\sqrt{\eta}\kappa_1\Delta}{(\kappa/2-i\Omega)^2+\Delta^2}Y_2\end{aligned}\tag{6a}$$

$$\begin{aligned}Y_\mathrm{out}(\Omega)=&\frac{2g\sqrt{\kappa_1}(\kappa/2-i\Omega)}{(\kappa/2-i\Omega)^2+\Delta^2}q+\frac{\kappa_1\Delta}{(\kappa/2-i\Omega)^2-\Delta^2}X_1-\frac{\sqrt{\eta}\kappa_1\Delta}{(\kappa/2-i\Omega)^2+\Delta^2}X_2\\&+\frac{(\frac{\eta-1}{2}\kappa_1-i\Omega)(\kappa/2-i\Omega)+\Delta^2}{(\kappa/2-i\Omega)^2+\Delta^2}Y_1-\frac{\sqrt{\eta}\kappa_1(\kappa/2-i\Omega)}{(\kappa/2-i\Omega)^2+\Delta^2}Y_2\end{aligned}\tag{6b}$$

For the intensity modulation readout mechanism [1] where the intensity of the optical field is detected while keeping the laser frequency locked on the side of the optical resonance, the detected photocurrent can be written as

$$i(\Omega)=|\alpha_\mathrm{out}|^2\delta(0)+|\alpha_\mathrm{out}|\cos\theta\cdot X_\mathrm{out}(\Omega)+|\alpha_\mathrm{out}|\sin\theta\cdot Y_\mathrm{out}(\Omega)\tag{7}$$

Here, $|\alpha_\mathrm{out}|=\sqrt{\frac{\Delta^2+\kappa_1^2(\eta-1)^2/4}{\Delta^2+\kappa_1^2(\eta+1)^2/4}N_\mathrm{in}}$ is the constant output field amplitude, which is set as 1 for normalization. $\delta(0)$ is the delta function, $\theta=\arctan(\frac{\mathrm{Im}(\alpha_\mathrm{out})}{\mathrm{Re}(\alpha_\mathrm{out})})$ is the phase angle of the output field. $\mathrm{Re}(\alpha_\mathrm{out})$ and $\mathrm{Im}(\alpha_\mathrm{out})$ denote the real and imaginary parts of the cavity field, respectively. With the DC term omitted, the PSD of the photocurrent can be expressed as:

$$\begin{aligned}S_{ii}(\Omega)&=S_\mathrm{thermal}(\Omega)+S_\mathrm{shot}(\Omega)+S_\mathrm{back-action}(\Omega)\\&=2A_1|\chi_\Delta|^2\gamma S_{p_\mathrm{in}p_\mathrm{in}}+S_{X_\mathrm{in}X_\mathrm{in}}+4A_1A_2\gamma|\chi_\Delta|^2|C_\mathrm{eff}^\Delta|S_{X_\mathrm{in}X_\mathrm{in}}\end{aligned}\tag{8}$$

Here, $A_1=\frac{4g^2\kappa_1[\Delta^2\cos^2\theta+(\frac{(\eta+1)^2}{4}\kappa_1^2+\Omega^2)\sin^2\theta-\frac{\eta+1}{2}\kappa_1\Delta\sin 2\theta]}{(\frac{(\eta+1)^2}{4}\kappa_1^2-\Omega^2+\Delta^2)^2+(\eta+1)^2\kappa_1^2\Omega^2}$, $A_2=\frac{(\frac{(\eta+1)^2}{4}\kappa_1^2+\Omega^2)(\Delta^2+\frac{(\eta+1)^2}{4}\kappa_1^2+\Omega^2)}{(\frac{(\eta+1)^2}{4}\kappa_1^2-\Omega^2+\Delta^2)^2+(\eta+1)^2\kappa_1^2\Omega^2}$. The first term in Eq. (8) is the thermal noise due to the heating of the thermal bath. The second term is the shot noise of the output light, which maintains $\frac{1}{2}$ for coherent light, regardless of the detuning. The third term originates from the radiation pressure shot noise heating, namely back-action noise. Figure S1a presents the PSDs of the thermal noise (green curve), shot noise (dashed black curve), back-action noise (blue curve), and total noise (red curve), using the parameters of our designed magnetometer as an example. It can be seen that the $S_\mathrm{back-action}$ is much smaller than the S_thermal and therefore is negligible. Near the mechanical resonance frequency, the S_thermal is much greater than the S_shot. In contrast, the S_shot is dominant in the off-resonance frequency ranges. We can then deduce the sensitivity of the magnetometer:

$$\begin{aligned}B_\mathrm{min}(\Omega)&=\frac{1}{c_\mathrm{act}}\sqrt{(\frac{S_\mathrm{thermal}}{A_1|\chi_\Delta|^2}+\frac{S_\mathrm{shot}}{A_1|\chi_\Delta|^2}+\frac{S_\mathrm{back-action}}{A_1|\chi_\Delta|^2})\cdot 2p_\mathrm{zp}^2}\\&=\frac{1}{c_\mathrm{act}}\sqrt{2m_\mathrm{eff}\gamma k_B\mathcal{T}+\frac{p_\mathrm{zp}^2}{A_1|\chi_\Delta|^2}+2A_2m_\mathrm{eff}\gamma\hbar\Omega_\mathrm{m}|C_\mathrm{eff}^\Delta|}\end{aligned}\tag{9}$$

Here, $c_\mathrm{act}=F/B$ is the magnetic actuation constant. According to Eq. (9), the sensitivity is determined by the sensor noise and the value of c_act. Since thermal noise is a fundamental noise source, its contribution to sensitivity only depends on the inherent parameters of the magnetometers and is independent of the measurement parameters. In contrast, the contribution of the shot noise to the sensitivity is dependent on the value of A_1, which is associated with the measurement parameters such as optical detuning (Δ), optical quality factor (Q_o), and incident power (P_in). Therefore, by optimizing the measurement parameters, we can increase the value of A_1 and thereby minimize the contribution of the shot noise to the sensitivity. For instance, the contribution of the shot noise to the sensitivity is inversely proportional to the incident power. Accordingly, increasing the incident power helps minimize the contribution of the shot noise to the sensitivity. In the experiment, we set the incident power at 40 µW, which is just below

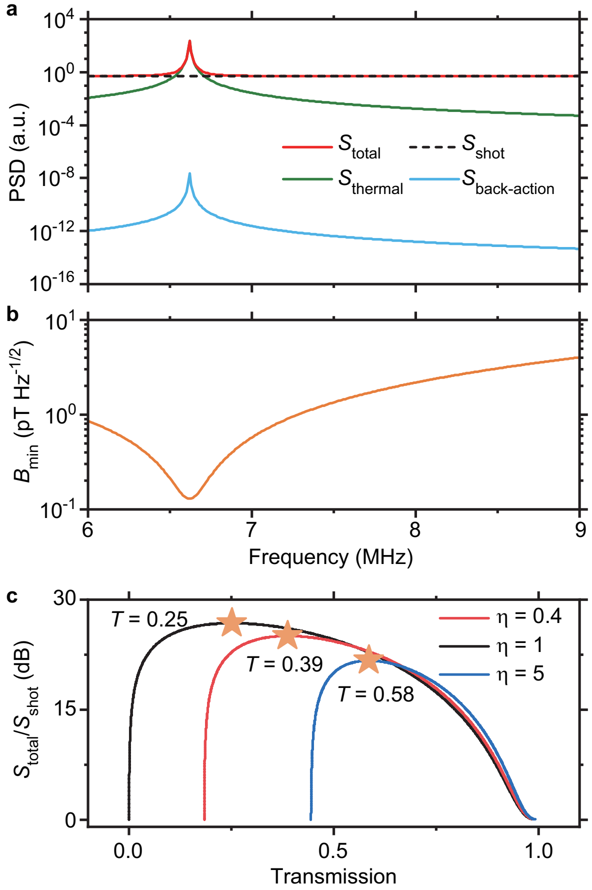

Fig. S 1. Theoretical calculation of our magnetometers. **a** Noise PSDs of the detected photocurrent. The red curve, green curve, dashed black curve, and blue curve are the calculated total noise, thermal noise, shot noise, and back-action noise, respectively. The magnetometer considered here has a radius of 305 µm, an RBM frequency of $\Omega_{\mathrm{m}}/2\pi$=6.62 MHz, a mechanical quality factor of $Q_{\mathrm{m}} = \Omega_{\mathrm{m}}/\gamma_{\mathrm{m}} = 800$, an optical quality factor of $Q_{\mathrm{o}} = \Omega_{\mathrm{o}}/\kappa_0 = 2.5 \times 10^6$, an effective mass of $m_{\mathrm{eff}} = 1.42$ µg, an optomechanical coupling strength of $G_{\mathrm{om}} = 3.64$ GHz/nm, and a magnetic actuation constant of $c_{\mathrm{act}} = 6.03$ N/T. The input laser power is set to 10 µW, with a detuning of $\Delta = \frac{\kappa}{2\sqrt{3}}$ (corresponding to a transmission $T = 1/4$) at the critical-coupled condition. **b** Sensitivity spectrum of the magnetometer. The parameters used here are the same as those in **a**. **c** Ratio of the total noise PSD to the shot noise PSD $S_{\mathrm{total}}/S_{\mathrm{shot}}$ at the frequency of the RBM, as a function of the transmission for the under-coupled ($\eta = 5$), critical-coupled ($\eta = 1$), and over-coupled ($\eta = 0.4$) conditions. Here the transmission T depends on the detuning Δ. η is defined as $\eta = \kappa_0/\kappa_1$, where κ_0 and κ_1 are the intrinsic and external energy decay rates of the optical mode. The parameters used here are the same as those in **a**.

the saturation power of the photodetector. Figure S1b illustrates the sensitivity spectrum of the magnetometer using the same parameters in Fig. S1a. The magnetic field sensitivity exhibits an optimal value of 129 fT Hz$^{-1/2}$ at the mechanical resonance frequency and degrades when it is far from the mechanical resonance.

Previous studies have derived the sensitivity that is under the condition of zero frequency detuning [2, 3], which can be expressed as:

$$B_{\mathrm{min}}(\Omega) = \frac{1}{c_{\mathrm{act}}}\sqrt{2m_{\mathrm{eff}}\gamma k_B \mathcal{T} + \frac{p_{\mathrm{zp}}^2(\kappa_1^2 + \Omega^2)}{4\kappa_1 g^2 |\chi|^2} + 2m_{\mathrm{eff}}\gamma\hbar\Omega_m|C_{\mathrm{eff}}|} \tag{10}$$

Here, the three terms under the square root are the thermal noise, shot noise, and back-action noise, respectively. In comparison to Eq. (10), the thermal-noise-dominant sensitivity in Eq. (9) remains unchanged. Conversely, the shot noise-dominant sensitivity is modified and is dependent on the detuning Δ. When the laser is blue-detuned ($\Delta > 0$), the energy decay rate of the mechanical mode γ is reduced. This results in an increase of the modified mechanical susceptibility χ_Δ and therefore a reduction of the contribution of shot noise to the total noise. When the laser is red-detuned ($\Delta < 0$), γ is increased, resulting in a decrease of χ_Δ and subsequently an increase of the contribution of shot noise to the total noise, which can deteriorate the sensitivity. Additionally, the value of Δ also affects A_1. As a result,

the effect of shot noise on sensitivity can be minimized by optimizing the transmission T, which is determined by the detuning Δ. We calculate the relationship between $S_{\mathrm{total}}/S_{\mathrm{shot}}$ and T, as a function of the transmission for under-coupled ($\eta = 5$), critical-coupled ($\eta = 1$), and over-coupled ($\eta = 0.4$) conditions. Figure S1c shows that $S_{\mathrm{total}}/S_{\mathrm{shot}}$ reaches its maximal value when T =0.58, 0.25, and 0.39, respectively. These transmission values correspond to the transmission of 1/4 of the minimum transmission, which can be expressed as $T = \frac{1}{4} \times (1 - T_{\mathrm{min}}) + T_{\mathrm{min}}$, with T_{min} denoting the minimum transmission. It means that the sensitivity can be optimized by tuning the laser frequency to the point where the transmission equals 1/4 of the minimum transmission for any coupling condition.

S2. Influence of optical quality factor on sensitivity

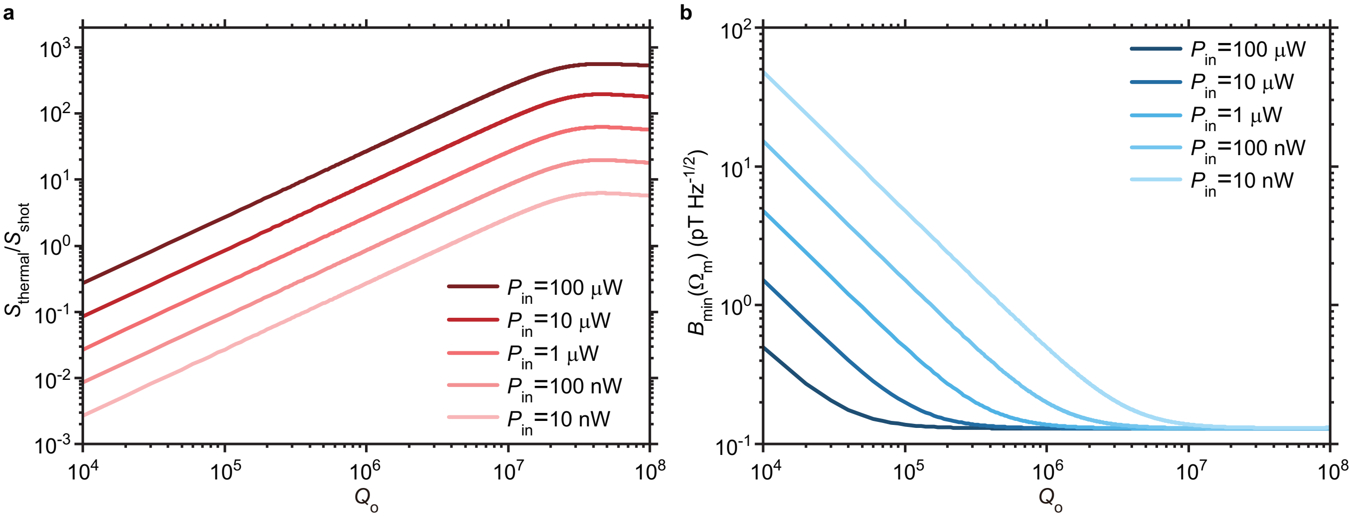

Fig. S 2. Influence of the optical quality factor on sensitivity. **a** Relationship between $S_{\mathrm{thermal}}/S_{\mathrm{shot}}$ and the optical quality factor at different incident laser powers. **b** Relationship between the sensitivity B_{min} and the optical quality factor at different incident laser powers. The parameters used here are the same as those in Fig. S1.

According to Eq. (9), the sensitivity of the magnetometer is limited by a combination of thermal noise and shot noise. The ratio of these two noise sources can be expressed as $S_{\mathrm{thermal}}/S_{\mathrm{shot}} = A_1$. Therefore, the sensitivity of the magnetometer can be optimized by optimizing the value of A_1. From the expression of A_1, we see that its value depends on the optical detuning (Δ), incident laser power (P_{in}), and intrinsic optical quality factor (Q_{o}). In Section 1, we analyzed how to minimize the contribution of shot noise by optimizing the optical detuning. In this part, we calculate the relationship between the magnetometer's sensitivity B_{min} and Q_{o} under the critical-coupled condition with different incident laser powers, as shown in Fig. S2. In the calculation, we take a magnetometer with a radius of 305 µm as an example and use the parameters consistent with Fig. S1. Figure S2a shows the relationship between $S_{\mathrm{thermal}}/S_{\mathrm{shot}}$ and Q_{o} at the mechanical resonance frequency for different P_{in}. At lower Q_{o} ($\kappa_1 \gg \Omega_{\mathrm{m}}$), $A_1 \propto P_{\mathrm{in}}/\kappa_1^2 = P_{\mathrm{in}} Q_{\mathrm{o}}^2$, thus $S_{\mathrm{thermal}}/S_{\mathrm{shot}}$ scales quadratically with Q_{o}. This indicates that the optical mode's response to mechanical displacement increases with Q_{o}. Once $S_{\mathrm{thermal}}/S_{\mathrm{shot}}$ exceeds 1, it enters the thermal-noise-dominant regime. Interestingly, the higher the Q_{o} is, the lower the P_{in} is required to reach the thermal-noise-limited regime. When Q_{o} is much greater than 3×10^7 ($\kappa_1 \ll \Omega_{\mathrm{m}}$), $A_1 \propto g^2\kappa_1 = P_{\mathrm{in}} \times \mathrm{constant}$, thus $S_{\mathrm{thermal}}/S_{\mathrm{shot}}$ reaches a plateau. This is because, although the optical mode's response to displacement decreases with Q_{o}, the optomechanical coupling strength increases, leading to a balance that maintains a constant ratio. Figure S2b shows $B_{\mathrm{min}}(\Omega_{\mathrm{m}})$ as a function of Q_{o}. At lower Q_{o}, the sensitivity increases linearly with the increase of Q_{o}. Once Q_{o} surpasses the transition point, the sensitivity reaches a plateau. Importantly, the higher the Q_{o} is, the lower the incident laser power P_{in} is required to achieve this sensitivity plateau. This allows the optimal sensitivity to be reached using lower optical powers, which is beneficial for reducing thermal effects in the magnetometer.

S3. BANDWIDTH OF THE MCOM

The MCOM magnetometer relies on dual resonances of both the optical and mechanical modes. Consequently, certain parameters like the optical quality factor (Q_{o}), incident laser power (P_{in}), optical detuning (Δ), and mechanical quality factor (Q_{m}), can affect the 3-dB sensing bandwidth of the device. Figure S3 shows the sensitivity spectra

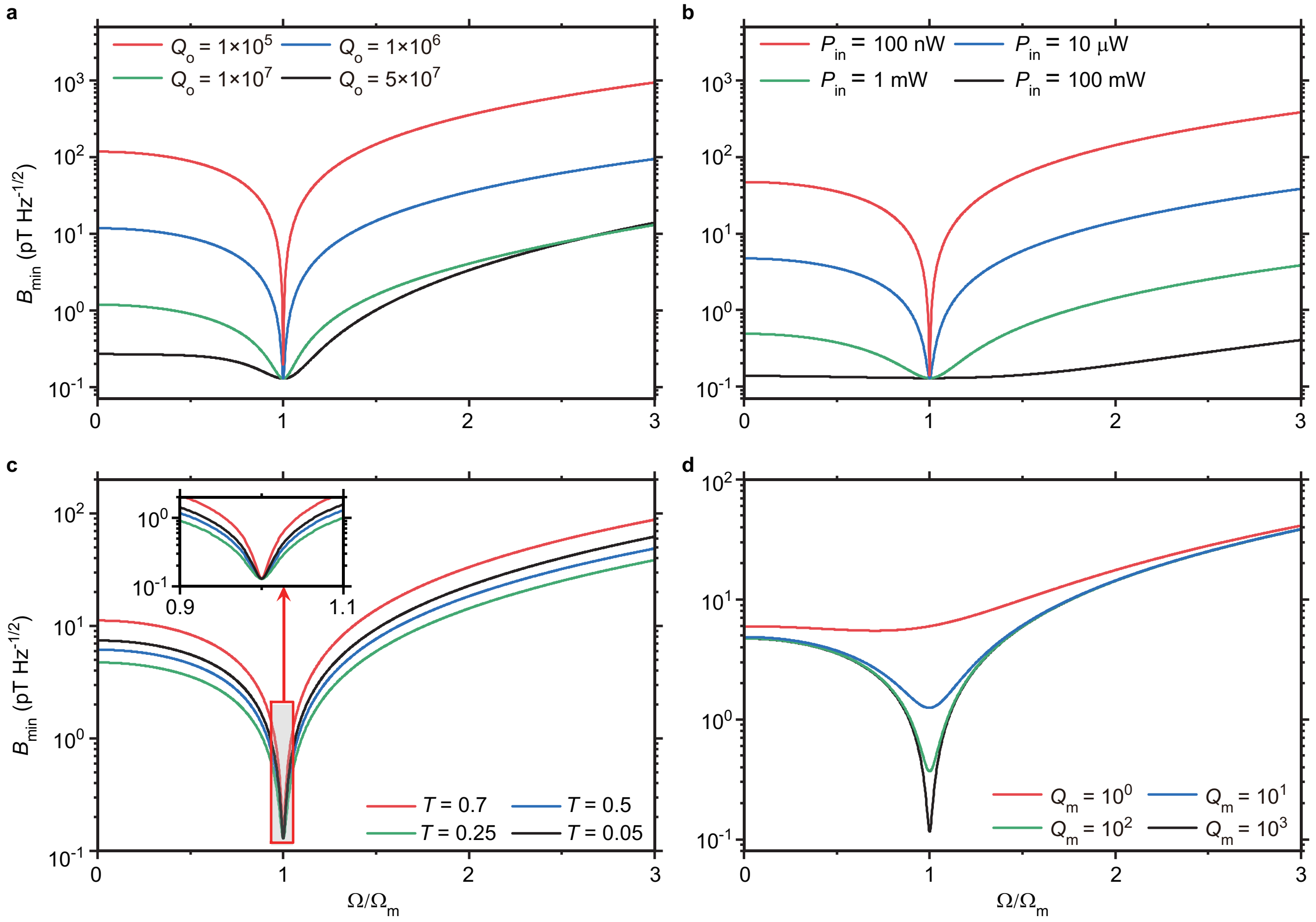

Fig. S 3. Sensitivity spectra under different optical and mechanical parameters. **a-d** Sensitivity spectra under different Q_o, P_in, T, and Q_m, respectively. The parameters used here are the same as those in Fig. S1.

under different parameters. As illustrated in Fig. S3a, the bandwidth of the magnetometer broadens as Q_o increases. Importantly, the sensitivity at the mechanical resonance frequency remains unchanged for Q_o greater than 1×10^5. When Q_o is increased to 5×10^7, the bandwidth is broadened to approximately the frequency of the mechanical mode. Figure S3b demonstrates that the bandwidth of the magnetometer broadens as P_in increases. Higher input power leads to a broader bandwidth, while the sensitivity at the mechanical resonance remains constant with increasing P_in. At an input power of 100 mW, the sensitivity spectrum becomes flat, and the bandwidth can reach three times the mechanical frequency. Figure S3c shows that when the transmission T equals 1/4, the magnetometer exhibits the largest bandwidth while maintaining the same on-mechanical-resonance sensitivity. Figure S3d reveals a trade-off between the sensitivity and bandwidth when varying Q_m. This means that a magnetometer with a higher Q_m has a better sensitivity but a narrower bandwidth.

S4. Transmission spectra with increasing magnetic field

To investigate the reduction in SNR when the magnetic field exceeds 2.56 µT, as shown in Fig. 3c in the main text, we conduct measurements of the transmission spectra of the optical mode under different magnetic fields. The measurement results are presented in Fig. S4. Due to the thermo-optic effect, the transmission of the optical mode exhibits a thermal triangle lineshape instead of a Lorentzian lineshape. Within the range of 2.56 nT to 768 nT, the shape of the transmission spectrum remains unchanged. However, with further increases in the magnetic field, the mechanical displacement of the optical microcavity induced by the magnetic field can no longer be considered as a perturbation. In the case of large displacement, cascaded n photon-phonon scattering events occur. Therefore, a large fraction of the intracavity photons at ω_o are scattered to higher frequencies, resulting in increased κ_0 and the emergence of additional dips in the transmission spectrum near each detuning $\Delta = n\Omega_\mathrm{m}$ [4–6]. However, discrete dips with frequency internal of Ω_m cannot be effectively recognized since the system is under the sideband-unresolved

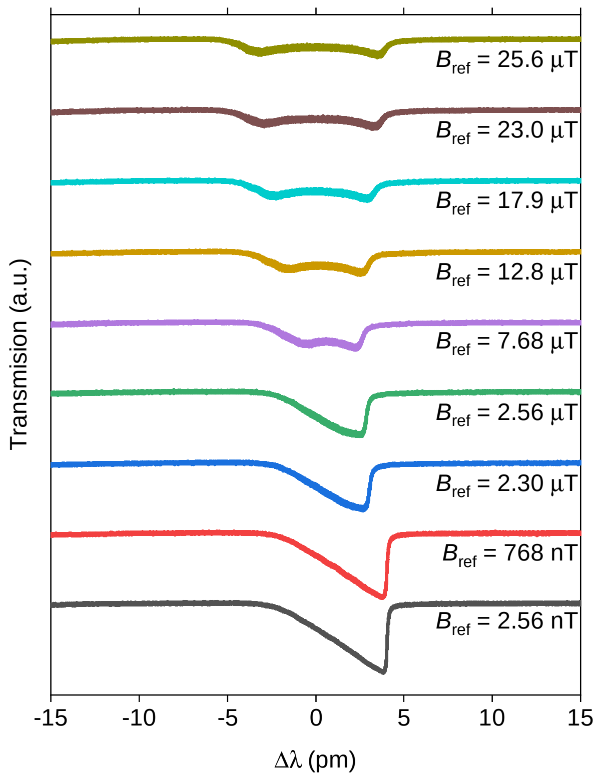

Fig. S 4. Transmission spectra with increasing magnetic field.

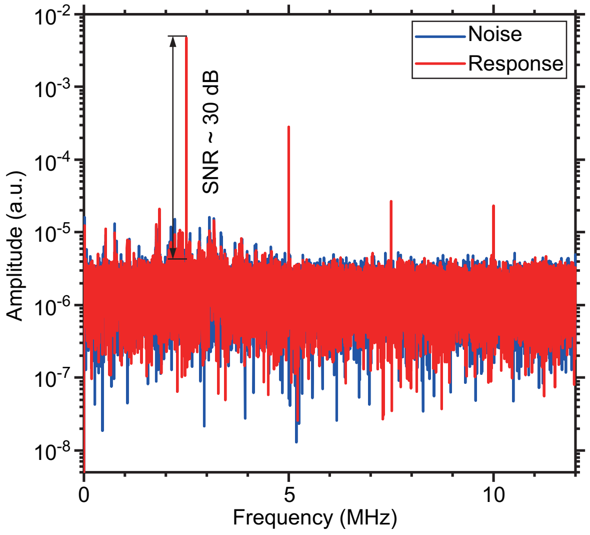

Fig. S 5. Frequency-domain response of the R=205 μm magnetometer with(with) and without(blue line) the pulsed signal shown in Fig. 5b.

regime ($\Omega_m > \kappa$), which leads to the overlap between different dips. In this case, the intracavity photon number at ω_o reduces accordingly due to the increased intrinsic decay rate of the optical mode. This weakens the readout ability of the mechanical motion, resulting in a reduction of the measured SNR.

S5. Analysis of the microcavity response to the pulsed magnetic signal

Using the fast Fourier transform, we convert the optical transmission response of the magnetometer (Fig. 5c in the main text) into the frequency domain, as shown in Fig. S5. The amplitude SNR is calculated to be around 30 dB at the repetition frequency of the original signal (2.5 MHz), corresponding to a minimum detectable magnetic field of 4.32 nT with an RBW of 1 kHz. Peaks at 5 MHz, 7.5 MHz, and 10 MHz are the cavity response to the high-frequency components of the corona current.

---

[1] Bowen, W. P. & Milburn, G. J. Quantum Optomechanics. (Boca Raton: CRC Press, 2015).
[2] Aspelmeyer, M., Kippenberg, T. J. & Marquardt, F. Cavity optomechanics. *Reviews of Modern Physics* **86**, 1391-1452 (2014).
[3] Bowen, W. P. & Yu, C. Q. Cavity optomechanical magnetometers. in High Sensitivity Magnetometers (eds Grosz, A., Haji-Sheikh, M. J. & Mukhopadhyay, S. C.) (Cham: Springer, 2017), 313-338.
[4] Hu, Y. et al. Generation of optical frequency comb via giant optomechanical oscillation. *Physical Review Letters* **127**, 134301 (2021).
[5] Schliesser, A. et al. Resolved-sideband cooling of a micromechanical oscillator. *Nature Physics* **4**, 415-419 (2008).
[6] Krause, A. G. et al. Nonlinear radiation pressure dynamics in an optomechanical crystal. *Physical Review Letters* **115**, 233601 (2015).
